# Cardiotrophin-like cytokine factor 1 forms a complex with IL12/IL23p40

**DOI:** 10.1038/s41598-025-08737-1

**Published:** 2025-09-30

**Authors:** Véronique Laplante, Marine Rousseau, Sonia Terki, Lara Abou Monsef, Rafael Najmanovich, Sylvie Lesage, Jean-François Gauchat, Sarah Pasquin

**Affiliations:** 1 Département de pharmacologie et physiologie, Université de Montréal, Montréal, Québec H3T 1J4 Canada; 2 Département de microbiologie, infectiologie et immunologie, Université de Montréal, Montréal, Québec H3T 1J4 Canada; 3 Centre de recherche de l’Hôpital Maisonneuve-Rosemont, Université de Montréal, Montréal, Québec H1T 4B3 Canada

**Keywords:** Cytokines, Immunology

## Abstract

Cardiotrophin-like cytokine factor 1 (CLCF1) is a cytokine of the IL6/IL12 superfamily with pro-neurotrophic and immune-modulating functions. Although the pro-neurotrophic activities of CLCF1 are mediated through the ciliary neurotrophic factor receptor (CNTFR), the α receptor chain of the CNTFR, CNTFRα, is not expressed by immune cells. This suggests the presence of an alternative receptor or protein complex that mediates the immune activities of CLCF1. Using the BioID2 proximity-dependent biotinylation assay, we identified p40, the β subunit of IL12/IL23, as a potential interaction partner for CLCF1. We confirmed the protein-protein interaction between CLCF1 and p40 using co-immunoprecipitation and a proximity ligation assay. We also observed that the CLCF1-p40 complex forms both intracellularly and in the extracellular space. Furthermore, secretion of the CLCF1-p40 heterodimer was induced by the cytokine receptor-like factor 1 (CRLF1), leading to the release of a tripartite CLCF1-CRLF1-p40 complex. Lastly, we showed that a CLCF1-p40 fusion protein binds to both CNTFRα and IL12Rβ1. Taken together, our results uncover a new putative composite cytokine of the IL6/IL12 superfamily, which might affect our understanding of both CLCF1 activities and p40-associated pathologies. Moreover, our results reinforce the connection between the IL6 and IL12 cytokine families, and suggest that other possible protein interactions between these two families should be further investigated.

## Introduction

The cardiotrophin-like cytokine factor 1 (CLCF1) is a cytokine of the IL6/IL12 superfamily^1^. CLCF1 mRNA can be detected in various immune organs, such as the bone marrow, spleen, and lymph nodes, as well as in the female reproductive organs (uterus and ovaries), and in the lungs^2,3^. Intracellular CLCF1 protein expression can also be detected by flow cytometry in naïve and activated mouse T cells, as well as B, NK and myeloid cells^4^. CLCF1 needs to form a complex with the cytokine receptor-like factor 1 (CRLF1) to be secreted^5^. CLCF1 activates the ciliary neurotrophic factor receptor (CNTFR), which is composed of three receptor chains : CNTFRα which, upon binding to CLCF1, recruits the signaling receptor chains leukemia inhibitory factor receptor β (LIFRβ) and gp130^5^. The CLCF1-CNTFR axis then activates the JAK/STAT3 signaling pathway^5,6^.

CLCF1 possesses a broad spectrum of biological activities. In mice, it plays important pro-neurotrophic functions for the embryonic development of motorneurons^7^ such that the complete knock-out of *Crlf1* or *Clcf1* in mice is lethal at P1^7–9^. Moreover, CLCF1 is involved in the circadian control of locomotor activity^10^. CLCF1-CRLF1 is also secreted by cancer-associated fibroblasts, and acts as a pro-oncogenic factor in a mouse model of non-small cell lung cancer^11^. Conversely, injections of CLCF1 in a mouse model of idiopathic pulmonary fibrosis leads to an accumulation of CD4^+^ T cells in lung tissues, accompanied by a decrease of pulmonary fibrosis^12^. CLCF1 binds lipoproteins^13^ and inhibits energy expenditure by supressing brown fat thermogenesis^14,15^. It also modulates mesenchymal cells osteoblastic differentiation^16^. Finally, injections or overexpression of CLCF1 in mice lead to B cell hyperplasia^3,17^, IgG and IgM hyperglobulinemia^3,17^, and increased numbers of circulating CD11b^+^ myeloid cells^18^.

While the neurotrophic, metabolic and oncogenic activities of CLCF1 are mediated through the CNTFR^7,19^ mRNA data shows that CNTFRα is not expressed by immune cells. We therefore hypothesize that the immune activities of CLCF1 might be achieved through an alternative CLCF1 composite cytokine recruiting a different cytokine receptor.

The IL6 and IL12 cytokine families share a close evolutionary relationship^20–22^. Indeed, the four alpha helix structure of CLCF1 and other IL6 family cytokines is homologous to the structure of the α subunits of the IL12 family^23,24^. On the other hand, CRLF1 is structurally homologous to the β subunits of the IL12 family and induces CLCF1 secretion in a way reminiscent of how p40 induces p35 and p19 secretion^23,24^. Accordingly, many protein-protein interactions between IL6 and IL12 family cytokines have been reported in the literature, such as CRLF1-p28^25,26^, CRLF1-p35^27^ and IL6-EBI3^28^. Therefore, we investigated whether CLCF1 could interact with other members of the IL6/IL12 superfamily.

To identify new protein-protein interactions for CLCF1, we used the BioID2 proximity-dependent biotinylation assay^29^ in HEK-293T cells which were co-transfected with candidate partners. We observed the biotinylation of p40, the β subunit of IL12/IL23 and a soluble receptor-like protein which is structurally related to CNTFRα^30^.

p40 is a cytokine subunit that binds with either p35 to form IL12^31^, or p19 to form IL23^32^. IL12 is mostly known for its role in the differentiation of CD4^+^ T cells into Type 1 helper T cells (Th1)^33^ whereas IL23 plays critical roles in many autoimmune diseases such as inflammatory bowel disease, psoriasis, and multiple sclerosis^34^. The pathological importance of p40-related cytokines has been highlighted by the growing use of p40-neutralizing antibodies, such as Ustekinumab^35,36^, in the clinic. Studies also show that p40 can be produced and secreted as a monomer or a homodimer^37,38^ which can inhibit the binding of IL12 and IL23 to the IL12Rβ1 receptor chain^37–39^. Furthermore, free p40 can also associate with p35 in the extracellular space to form *de novo* IL12^40^.

Following the results of our BioID2 assay, we confirmed the protein-protein interaction between CLCF1 and p40 using both co-immunoprecipitation experiments and a proximity ligation assay (PLA). We further showed that p40 could not drive the secretion of CLCF1, and that the addition of CRLF1 allowed for the secretion of a tripartite CLCF1-CRLF1-p40 protein complex. Altogether, our results identify a new composite cytokine complex in the IL6/IL12 superfamily, which could help further our understanding of CLCF1 immune activities and shed a new light on p40-driven autoimmune pathologies.

## Results

### CLCF1 forms a protein complex with p40, the β subunit of IL12/IL23

**Fig. 1:**
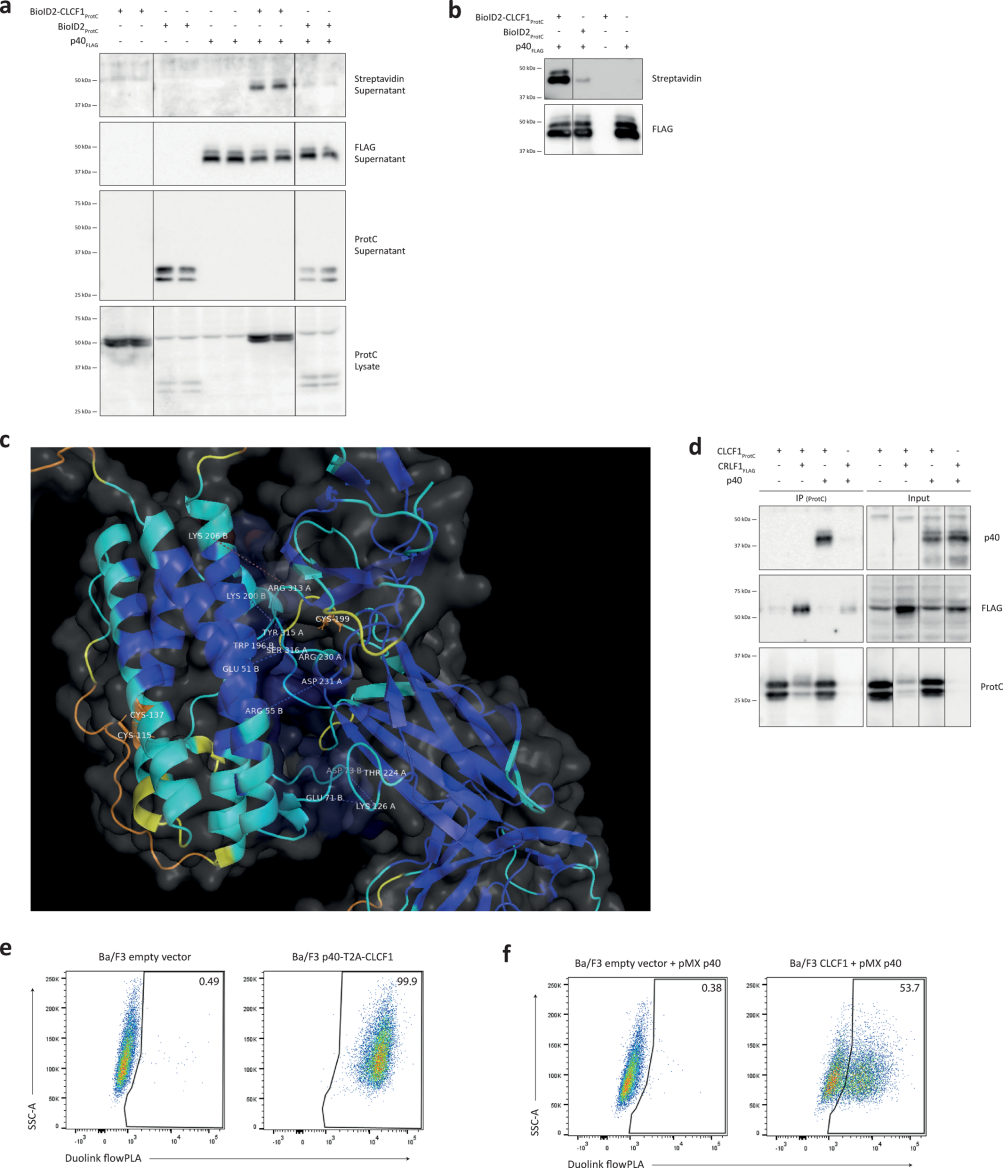
CLCF1 forms a complex with the β subunit of IL12/IL23 (p40). (**a-b**) HEK-293T cells were transfected with indicated combinations of pcDNA3.4 derivatives coding for p40_FLAG_, BioID2_ProtC_ or BioID2-CLCF1_ProtC_. (**a**) Western blot analysis of cell lysates and supernatants. (**b**) Cell supernatants were subjected to immunoprecipitation with anti-FLAG affinity gel. (**a–b**) Membranes were probed with anti-FLAG or anti-ProtC to confirm protein expression, or streptavidin to detect proximity-dependent biotinylation of co-transfected proteins. Blots are representative of 3 separate experiments. (**c**) p40 (chain A) is predicted to interact with CLCF1 (chain B) with a ΔG of -16.40 kcal/mol. The protein regions are colored according to the accuracy of the predicted model, with brighter shades of blue representing the highest confidence. Residues predicted to be implicated in the interaction interface are annotated for each protein. Pairwise interactions are denoted by dashed lines connecting the Cα atoms of the respective residues but represent the sum of all pairwise atomic interactions between the atoms in the main or side chain of the two residues. Cysteine residues are marked in orange. (**d**) Cell lysates from HEK-293T cells transfected with indicated combinations of pcDNA3.4 derivatives coding for CLCF1_ProtC_, CRLF1_FLAG_ or p40 were subjected to immunoprecipitation with anti-ProtC affinity matrix. Membranes were probed with anti-FLAG, anti-ProtC or anti-p40. Blots show eluted proteins (IP) or proteins prior to immunoaffinity chromatography (Input). Blots are representative of 3 separate experiments. (**a**,** b**,** d**) Western blot images were cropped, and original blots are presented in Supplementary Figure S2. (**e-f**) The Duolink flowPLA proximity ligation assay was used to detect protein interaction between CLCF1 and p40 in Ba/F3 cells transduced with pMX retroviruses coding for (**e**) p40-T2A-CLCF1 cDNA, or (**f**) CLCF1 and p40 cDNA. Figures are representative of (**e**) 2 or (**f**) 4 separate experiments.

To better understand the action mechanism of CLCF1 immune activities, we searched for new CLCF1 interaction partners using the BioID2 proximity-dependent biotinylation assay^29^. This technique uses a biotin ligase from *A. aeolicus* that was mutated to allow the promiscuous biotinylation of nearby proteins. Thus, it allows the labelling and detection of potential interaction partners when a protein of interest, used as bait, is fused with the mutated biotin ligase^29^. In our case, we used a ProtC-tagged BioID2-CLCF1 fusion protein, which we co-expressed in HEK-293T cells with FLAG-tagged candidate partners. We observed a biotinylated protein in the supernatant of HEK-293T cells that were co-transfected with the BioID2-CLCF1 fusion protein and FLAG-tagged p40 (Fig. 1a). The SDS-PAGE migration profile of this biotinylated protein was consistent with the one observed for p40 using the anti-FLAG mAb. To confirm that p40 is indeed biotinylated, we performed anti-FLAG immunoaffinity chromatography to pull down p40 from the cell supernatant. We observed that the biotinylation level of p40 in the presence of the BioID2-CLCF1 fusion protein was much greater than the non-specific biotinylation caused by BioID2 alone (Fig. 1b). These results point to a possible interaction between CLCF1 and p40. To further confirm whether a CLCF1-p40 interaction is possible, we generated in silico a model of the protein complex (Fig. 1c). The resulting minimized complex is predicted to have a favorable ΔG of -16.40 kcal/mol (i.e. K_D_ ≈ 0.96 pM), which suggests that CLCF1 and p40 could form a high-affinity complex. Notably, SER16-GLU51, ASP231-ARG55 and TYR315-TRP196 (p40-CLCF1) appear to be amongst the top contributing interactions. A full list of the predicted interactions is available in Supplementary Table S5.

To better investigate the formation of a complex between CLCF1 and p40, we co-expressed ProtC-tagged CLCF1 with untagged p40 in HEK-293T cells, and immunoprecipitated CLCF1 from the cell lysate with anti-ProtC immunoaffinity chromatography. Our results show that p40 could be co-immunoprecipitated with CLCF1 from the lysate of HEK-293T cells (Fig. 1d), therefore confirming a protein-protein interaction between CLCF1 and p40. FLAG-tagged CRLF1, the secretion partner for CLCF1, was used as positive control.

To further confirm the interaction between CLCF1 and p40, we used a Duolink proximity ligation assay, which utilizes oligonucleotides bound to secondary antibodies indirectly targeting CLCF1 and p40. When in proximity, these oligonucleotides can be hybridized to a connector oligonucleotide and ligated to form a circular template that is then subjected to rolling-circle amplification and detected with a fluorescent detection probe. We first used this technique in Ba/F3 cells co-expressing CLCF1 and p40 from a bicistronic cDNA in which they were connected by a T2A “auto-cleavable” peptide^41^ (p40-T2A-CLCF1). A strong positive PLA signal could be detected by flow cytometry in these cells (Fig. 1e), thus confirming the protein-protein interaction between CLCF1 and p40. To show that this signal was not due to contaminating p40-CLCF1 fusion protein caused by an incomplete “cleavage” at the T2A sequence, we transduced a p40 cDNA into Ba/F3 cells that stably expressed CLCF1. The proximity ligation assay in these cells showed a positive PLA signal in about 55% of cells (Fig. 1f), which is consistent with the percentage of p40-expressing cells detected by intracellular staining (Supplementary Fig. S1). Altogether, these experiments revealed the formation of a complex between CLCF1 and the β subunit of IL12/IL23.

### CLCF1, CRLF1 and p40 form a secreted tripartite complex

**Fig. 2:**
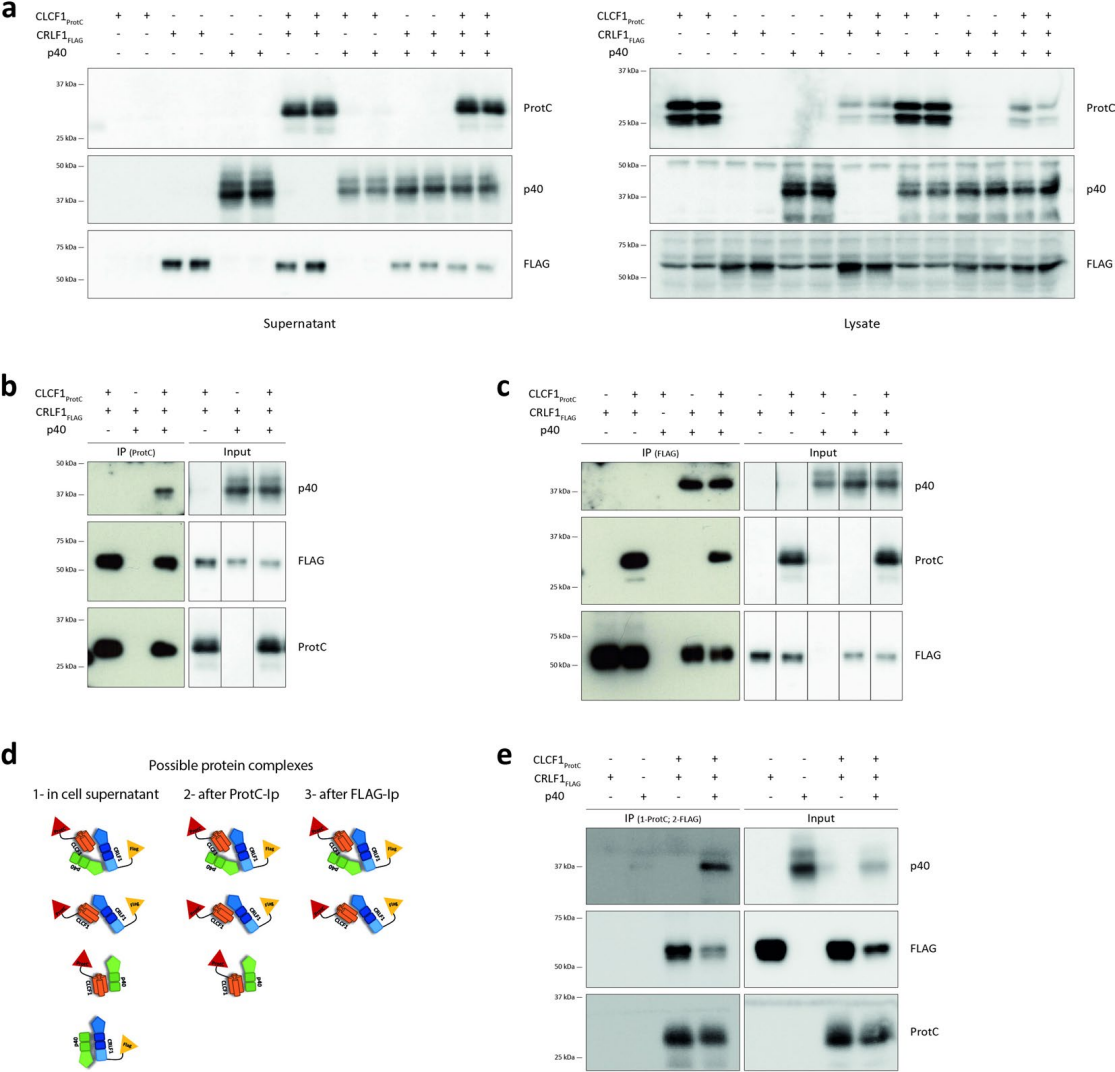
CLCF1, CRLF1 and p40 form a secreted tripartite complex. (**a-c**,** e)** HEK-293T cells were transfected with indicated combinations of pcDNA3.4 derivatives coding for CLCF1_ProtC_, CRLF1_FLAG_ or p40. (**a**) Western blot analysis of cell supernatants (left panel) and lysates (right panel). (**b**, **c**,** e**) Cell supernatants were subjected to (**b**) simple immunoprecipitation with anti-ProtC affinity matrix, (**c**) simple immunoprecipitation with anti-FLAG affinity gel, or (**e**) sequential immunoprecipitation with anti-ProtC affinity matrix followed by anti-FLAG affinity gel. (**d**) Possible protein complexes at each step of the sequential immunoprecipitation experiment are represented schematically. (**a-c**,** e**) Membranes were probed with anti-ProtC, anti-FLAG or anti-p40. Blots show eluted proteins (IP) or proteins prior to immunoaffinity chromatography (Input). Western blot images were cropped, and original blots are presented in Supplementary Figure S3. Blots are representative of 3–5 experiments.

CLCF1 is secreted as a complex with CRLF1^5^ or the soluble form of CNTFRα^42^. Similarly, p40 forms complexes with p35 and p19 to drive their secretion in the form of the composite cytokines IL12 and IL23^31,32^. Interestingly, CNTFRα and p40 are structurally related^43^. Therefore, we investigated whether p40 could be an alternative secretion partner for CLCF1. No CLCF1 could be detected in the supernatant of HEK-293T cells co-expressing CLCF1 and p40 (Fig. 2a), indicating that p40 cannot substitute CRLF1 or CNTFRα to drive the secretion of CLCF1. However, we observed that CRLF1 could induce the secretion of CLCF1 in the presence of p40 (Fig. 2a), suggesting that p40 and CRLF1 bind different sites of CLCF1.

We further investigated whether the presence of CRLF1 interferes with the formation of the CLCF1-p40 complex. To do so, we co-transfected ProtC-tagged CLCF1, FLAG-tagged CRLF1 and untagged p40 cDNA in HEK-293T cells, and immunoprecipitated CLCF1 in the cell supernatant. Both CRLF1 and p40 co-immunoprecipitated with CLCF1 (Fig. 2b), which confirms the presence of both CLCF1-p40 and CLCF1-CRLF1 complexes in the supernatant of co-transfected HEK-293T cells. Unexpectedly, we observed that isolation of CRLF1 from the supernatant of HEK-293T cells allowed the co-immunoprecipitation of p40 even in the absence of CLCF1 cDNA (Fig. 2c), suggesting that CRLF1 can bind p40 independently of CLCF1. Altogether, these results indicate the formation of three protein complexes : the expected CLCF1-CRLF1 complex, as well as the previously unknown CLCF1-p40 and CRLF1-p40 complexes.

We next investigated whether CLCF1, CRLF1 and p40 could form a tripartite CLCF1-CRLF1-p40 protein complex. We subjected the culture medium from HEK-293T cells co-expressing ProtC-tagged CLCF1, FLAG-tagged CRLF1 and untagged p40 to sequential immunoprecipitations. We first immunoprecipitated CLCF1 to isolate CLCF1-p40 and CLCF1-CRLF1 complexes, as well as the potential CLCF1-CRLF1-p40 complexes from the samples. We subsequently immunoprecipitated CRLF1 to exclude the CLCF1-p40 complexes (Fig. 2d). Following these sequential immunoprecipitations, we observed the co-immunoprecipitation of p40 with CLCF1 and CRLF1 (Fig. 2e), thus confirming the formation of a tripartite complex between CLCF1, CRLF1 and p40.

### CLCF1 and p40 interact in the extracellular space

**Fig. 3:**
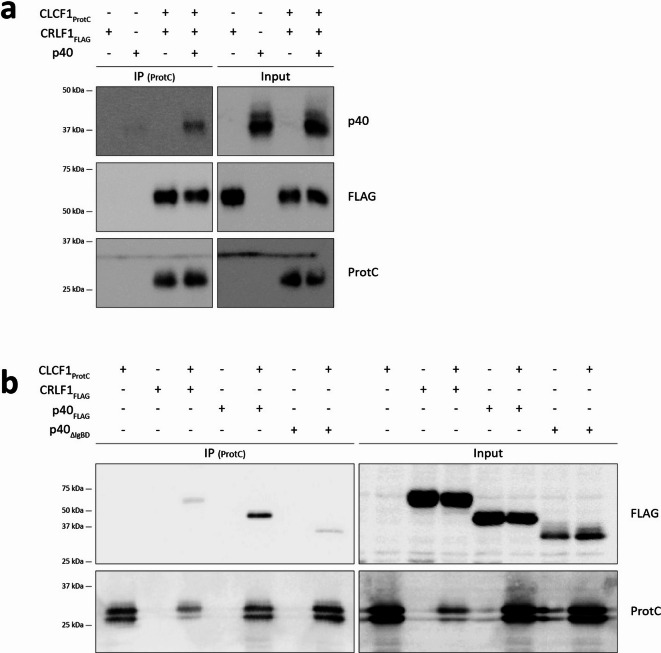
The CLCF1-p40 complex can form in the extracellular space and does not require the immunoglobulin binding domain of p40. (**a**) Cell supernatants from HEK-293T cells transfected with pcDNA3.4 derivatives coding for CLCF1_ProtC_ and CRLF1_FLAG_, or p40 were mixed in equal parts, incubated at 37 °C, and subjected to immunoprecipitation with anti-ProtC affinity matrix. Blots are representative of 5 separate experiments. (**b**) Cell lysates from HEK-293T cells transfected with indicated combinations of pcDNA3.4 derivatives coding for CLCF1_ProtC_, CRLF1_FLAG_, p40_FLAG_ or p40_ΔIgBD_ were subjected to immunoprecipitation with anti-ProtC affinity matrix. Blots are representative of 2 separate experiments. (**a**, **b**) Membranes were probed with anti-ProtC, anti-FLAG or anti-p40. Blots show eluted proteins (IP) or proteins prior to immunoaffinity chromatography (Input). Western blot images were cropped, and original blots are presented in Supplementary Figure S4.

As we observed that the CLCF1-p40 complex could not be secreted from cells in the absence of CRLF1, and as both CLCF1 and p40 have been detected in the serum^14,44,45^, we investigated whether this complex could form in the extracellular space. To do so, we transfected p40 and CLCF1 cDNA separately in HEK-293T cells. CLCF1 was co-transfected with CRLF1 cDNA to allow its secretion. We then mixed and incubated the cell supernatants at 37 °C for 24 h to allow the formation of protein complexes, and analysed them by immunoaffinity chromatography. We observed that p40 could co-immunoprecipitate with CLCF1 (Fig. 3a), indicating that CLCF1 and p40 can interact in the extracellular space.

### p40 does not interact with CLCF1 through its immunoglobulin binding domain

We next sought to investigate which p40 domain is required for the formation of the CLCF1-p40 heterodimer. p40 comprises three fibronectin type III (FNIII) domains, which make up two potential cytokine binding domains: an immunoglobulin binding domain (IgBD) and a cytokine-binding homology region (CHR)^46^. p35 and p19 have both been shown to interact with the CHR of p40 to form IL12 and IL23 respectively^47^. To analyse which region of p40 CLCF1 binds to, we generated a cDNA deletion mutant coding for a FLAG-tagged p40 protein without its IgBD (p40_ΔIgBD_). We then co-expressed this p40_ΔIgBD_ protein with Prot-C tagged CLCF1 in HEK-293T cells, and subjected the cell lysates to anti-ProtC immunoaffinity chromatography. Our results showed that both p40 and p40_ΔIgBD_ could co-immunoprecipitate with CLCF1 (Fig. 3b), therefore suggesting that, like p35 an p19, CLCF1 interacts with the CHR of p40 rather than its IgBD. As CLCF1 is presumed to bind the IgBD of CRLF1^48^, our results suggest that CLCF1 can form a tripartite complex by binding p40 CHR and CRLF1 IgBD.

### The CLCF1-p40 complex binds CNTFRα and IL12Rβ1, and partially activates the CNTFR

**Fig. 4:**
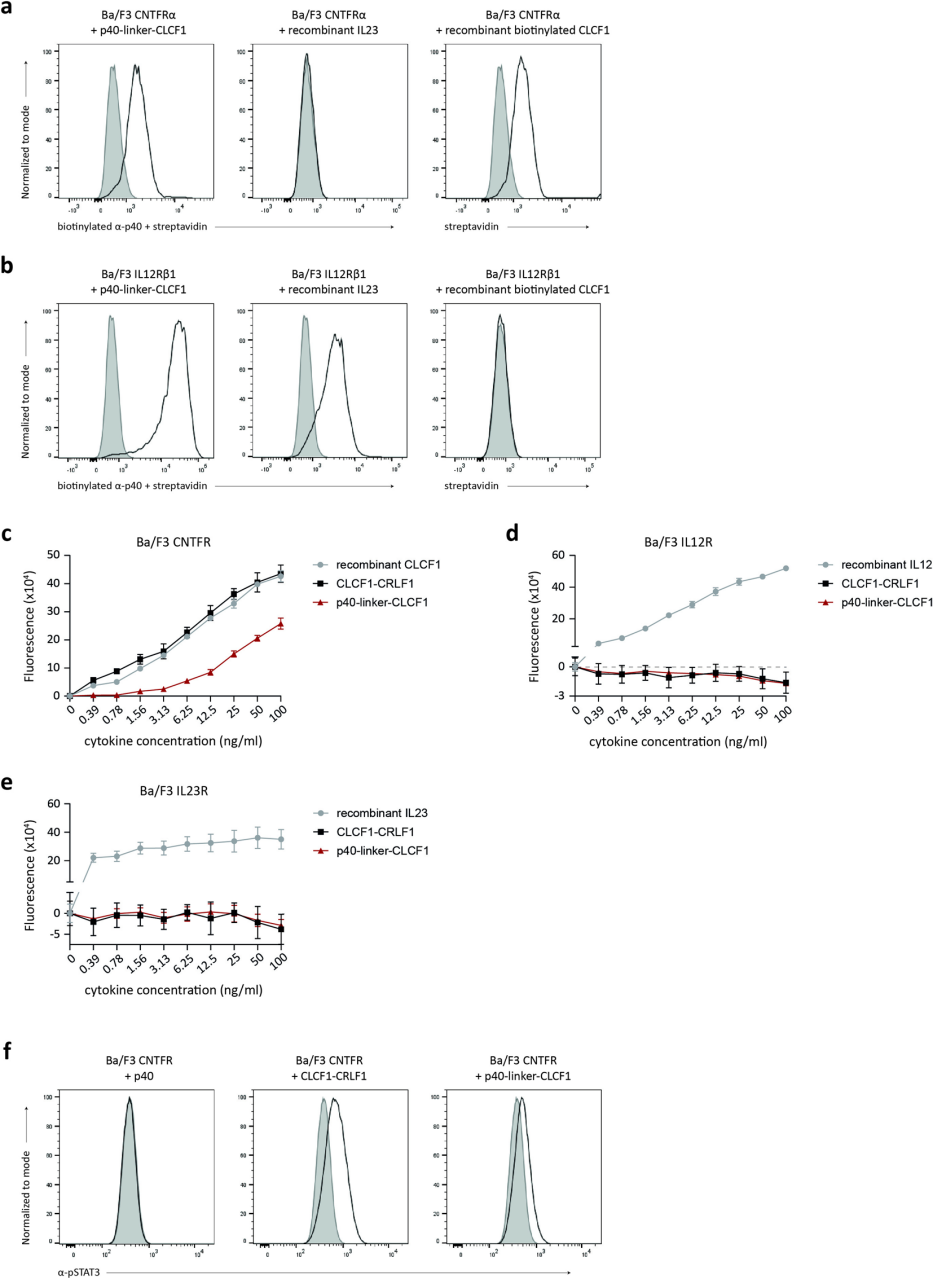
A p40-linker-CLCF1 fusion protein binds to both CNTFRα and IL12Rβ1, and retains partial activity on the CNTFR. (**a-b**) Ba/F3 cells were transduced with pMX derivatives coding for (**a**) CNTFRα or (**b**) IL12Rβ1. Cells were treated with p40-linker-CLCF1 fusion protein, recombinant IL23, or recombinant biotinylated CLCF1 (black line histogram on indicated graphs). Cytokine binding to cell surface receptors was detected using biotinylated anti-p40 followed by streptavidin for p40-linker-CLCF1 and recombinant IL23, or streptavidin alone for recombinant biotinylated CLCF1. Fluorescence signal of streptavidin background binding in the absence of cytokines, with or without biotinylated anti-p40, was used as negative control (filled grey histogram). Figures are representative of 3 separate experiments. (**c–e**) Ba/F3 cells were transduced with pMX retroviruses coding for the receptor chains of (**c**) CNTFR (CNTFRα, LIFRβ and gp130), (**d**) IL12R (IL12Rβ1 and IL12Rβ2), or (**e**) IL23R (IL12Rβ1 and IL23R). Proliferation of Ba/F3 cells in response to increasing concentrations of p40-linker-CLCF1 fusion protein (red triangles), or CLCF1-CRLF1 (black squares) was assessed. Recombinant CLCF1, IL12 or IL23 were used as positive controls where appropriate (grey circles). Ba/F3 proliferation was measured using the alamarBlue fluorometric assay. Figures are representative of 3–5 separate experiments run in triplicate culture wells. (**f**) STAT3 tyrosine phosphorylation in Ba/F3 cells expressing CNTFR was measured in response to p40-linker-CLCF1, CLCF1-CRLF1 or p40 stimulation (black line histogram on indicated graphs). Ba/F3 CNTFR cells were stimulated with an equal volume of concentrated cell supernatant from HEK-293T cells transfected with a pcDNA3.4 derivative coding for eGFP to detect baseline STAT3 tyrosine phosphorylation levels (filled grey histograms). Figures are representative of 3 separate experiments.

Finally, we evaluated how the interaction between CLCF1 and p40 affected the known properties of each individual cytokine. For this, we produced a p40-linker-CLCF1 fusion protein in HEK-293T cells to avoid CLCF1-p40 complex dissociation during the experimental protocols. We used Ba/F3 cells expressing either CNTFRα or IL12Rβ1, which are known to bind CLCF1 and p40 respectively^30,42^, to analyse the binding of the p40-linker-CLCF1 fusion protein to these receptor chains by flow cytometry. Recombinant CLCF1 and IL23 were used as positive and negative controls interchangeably for each receptor chain. Our results show that the p40-linker-CLCF1 fusion protein can bind to both CNTFRα (Fig. 4a) and IL12Rβ1 (Fig. 4b), which suggest that the interaction between CLCF1 and p40 does not prevent the binding of CLCF1 to CNTFRα, or of p40 to IL12Rβ1.

We next assessed the activity of the p40-linker-CLCF1 fusion protein on the CNTF, IL12 and IL23 receptors. To do so, we generated Ba/F3 derivatives that expressed either the CNTFR (CNTFRα, LIFRβ and gp130), IL12R (IL12Rβ1 and IL12Rβ2), or IL23R (IL12Rβ1 and IL23R). As expected, stimulation of these cells with recombinant CLCF1 (Fig. 4c), IL12 (Fig. 4d) or IL23 (Fig. 4e) respectively resulted in significant proliferation. We also showed that CLCF1-CRLF1 complexes produced in HEK-293T cells induced comparable levels of proliferation in Ba/F3 cells expressing CNTFR as control recombinant CLCF1 (Fig. 4c). Interestingly, we observed a reduced proliferation of Ba/F3 cells expressing CNTFR in response to the p40-linker-CLCF1 fusion protein compared to the response generated by CLCF1-CRLF1 or recombinant CLCF1 (Fig. 4c). This suggests that the interaction between CLCF1 and p40 partially inhibits the activity of CLCF1 on its receptor. Accordingly, we observed a reduction in the phosphorylation of STAT3 downstream of the CNTFR after stimulation with the p40-linker-CLCF1 fusion protein in comparison with CLCF1-CRLF1 stimulation (Fig. 4f). Ba/F3 cells expressing the CNTFR were also stimulated with p40, which was used as a negative control (Fig. 4f). On the other hand, p40-linker-CLCF1 fusion protein failed to stimulate the proliferation of Ba/F3 derivatives expressing IL12R (Fig. 4d) or IL23R (Fig. 4e), indicating that the biological activity of the CLCF1-p40 composite cytokine is distinct from those of IL12 and IL23.

## Discussion

Although CLCF1 possesses many immune activities, their action mechanism remains largely elusive as the known recruiting receptor chain for CLCF1, CNTFRα, is not expressed by immune cells. We hypothesized that CLCF1 immune activities might be achieved through unidentified CLCF1 composite cytokines, which could activate alternative immune cytokine receptors. We used the BioID2 proximity-dependent biotinylation assay to search for potential cytokine subunits that could associate with CLCF1. With this targeted method, we observed the biotinylation of p40, the β subunit of IL12/IL23, in the presence of our BioID2-CLCF1 bait construct. We confirmed the interaction between CLCF1 and p40 using co-immunoprecipitation and a proximity ligation assay. These results suggest that the BioID2-CLCF1 assay could be used in large scale proteomic studies to identify other potential interaction partners or receptors for CLCF1.

As p40 is structurally similar to CNTFRα^43^, which can drive CLCF1 secretion^42^, we investigated whether p40 could likewise induce the secretion of CLCF1. While we failed to detect a p40-induced secretion of CLCF1, we did show that CLCF1-p40 complexes are efficiently secreted in the presence of CRLF1. These results suggest that CRLF1 and p40 bind to CLCF1 through different sites. Unexpectedly, we also showed that CRLF1 forms a protein complex with p40 independently of CLCF1. Future studies on the biological role and impact of the CRLF1-p40 complex are warranted. Nonetheless, these interactions raise the possibility that CLCF1, CRLF1 and p40 form a heterotrimeric complex. A sequential co-immunoprecipitation protocol allowed us to confirm the formation of CLCF1-CRLF1-p40 tripartite complexes. To the best of our knowledge, this is the first observation of a trimeric composite cytokine.

We further observed that CLCF1-CRLF1 secreted from one cell and p40 secreted from another cell can associate in the extracellular space to form CLCF1-p40 complexes. As both CLCF1 and p40 can be detected in the serum^14,44,45^, it would be of interest to investigate whether the CLCF1-p40 complex is present in circulation, and whether its levels are affected by inflammatory or auto-immune diseases in which targeting p40 is therapeutically effective. Monoclonal antibodies against p40 are used for the treatment of psoriasis and Crohn’s disease^35,36^. The identification of a novel composite cytokine involving p40 invites further investigation regarding the biological impact of CLCF1-p40 complexes on p40-driven diseases, and how the CLCF1-p40 composite cytokine is affected by p40 therapeutic antibodies.

Finally, we investigated the impact of the CLCF1-p40 complex on the capacity of each individual protein to interact with their known receptors. While we showed that the CLCF1-p40 composite cytokine retained the ability to bind both CNTFRα and IL12Rβ1, we observed only a partial activation of the CNTFR, and no activation of either IL12R or IL23R in response to the CLCF1-p40 complex. These results indicate that p40 reduces the activation of CNTFR by CLCF1, and could therefore have partial antagonist functions. These are of potential relevance for CLCF1 physiological roles in neuronal development^7^, circadian rhythm^10^, thermogenesis^14,15^, and pathological roles in cancer^11,49^ or focal segmental glomerulosclerosis^50^. Our results also indicate that although the CLCF1-p40 complex binds the IL12Rβ1 subunit of IL12R and IL23R, it cannot replace p35 or p19 to activate these receptors, thus suggesting that the functions of the CLCF1-p40 composite cytokine are distinct from those of other p40-cytokines. Further studies are warranted to determine whether the new CLCF1-p40 composite cytokine is an IL12/IL23 antagonist, and for the identification of a receptor or biological activities specific to CLCF1-p40.

In conclusion, our results have uncovered the formation of new protein complexes between subunits of the IL6/IL12 superfamily, namely the CLCF1-p40 and CRLF1-p40 heterodimeric complexes, and the heterotrimeric CLCF1-CRLF1-p40 complex. These new protein complexes reinforce the connection between the IL6 and IL12 cytokine families, and join a growing list of known protein interactions between members of these two cytokine families. Further studies of the complexes biological activities could significantly increase our understanding of both CLCF1- and p40-driven pathologies.

## Methods

### Generation of plasmids

Codon optimized synthetic cDNA coding for human CLCF1 tagged with the protein C epitope (ProtC; EDQVDPRLIDGK) (CLCF1_ProtC_), human CRLF1 tagged with the FLAG epitope (DYKDDDDK) (CRLF1_FLAG_), human p40 untagged (p40) or tagged with the FLAG epitope (p40_FLAG_), truncated human p40 without its immunoglobulin-binding domain and tagged with the FLAG epitope (p40_ΔIgBD_), human p40_FLAG_-T2A-CLCF1_ProtC_ (p40-T2A-CLCF1)^51^ or human p40_FLAG_-(3xSGGGG) linker-CLCF1_ProtC_ fusion protein (p40-linker-CLCF1) were generated by GeneArt (Thermo Fisher Scientific, Burlington ON). A cDNA coding for the ApoE signal peptide fused with the *A. aeolicus* BioID2 biotin ligase mutant tagged with the protein C epitope (BioID2_ProtC_), or its derivative linked to the mature human CLCF1 (BioID2-CLCF1_ProtC_) were obtained from GeneArt. All cDNA were subcloned into the pcDNA3.4 vector (GeneArt) and/or the pMX retroviral expression vector^52^. Standard molecular biology methods were used to generate all plasmids.

## Generation of transgenic Ba/F3 cell lines

Cells from the IL3-dependent murine pro-B cell line Ba/F3 (Creative Bioarray, Shirley NY) were transduced in the presence of polybrene (8 µg/ml) using VSV-G pseudo-typed retroviruses coding for human IL12Rβ1, CNTFRα, LIFRβ, gp130, IL12Rβ2, IL23R, p40, CLCF1, p40-T2A-CLCF1, or p40-linker-CLCF1. Stable transgenic Ba/F3 derivatives expressing a single construct were selected with puromycin (1 µg/ml). Ba/F3 cells expressing CNTFRα, LIFRβ and gp130 (CNTFR) were positively selected using recombinant human CLCF1 produced in-house^13^. Ba/F3 cells expressing IL12Rβ1 and IL12Rβ2 (IL12R) or IL12Rβ1 and IL23R (IL23R) were positively selected using recombinant human IL12 and IL23 respectively (both from PeproTech, Cranbury NJ). For regular subculturing, Ba/F3 cells were expanded with recombinant murine IL3 (10 ng/ml; PeproTech) in RPMI 1640 medium supplemented with fetal bovine serum (FBS; 10%), L-glutamine (4 mM), penicillin (100 U/ml), streptomycin (100 µg/ml), HEPES pH 7.4 (10 mM) and β-mercaptoethanol (0.05 mM) (complete RPMI medium).

## Transient transfection of HEK-293T cells

HEK-293T cells (ATCC, Manassas VA) were maintained in DMEM medium supplemented with FBS (10%), penicillin (100 U/ml), streptomycin (100 µg/ml) and L-glutamine (4 mM) (complete DMEM medium). HEK-293T cells were transfected for 5 h in Opti-MEM medium (Thermo Fisher Scientific) with pcDNA3.4 derivatives (4 µg) and polyethylenimine. A pcDNA3.4 derivative coding for eGFP was used as negative control. Transfected cells were incubated in complete DMEM medium overnight, and in serum-free Opti-MEM medium for 3 d. Cell supernatant was collected, and cells were lysed in cell lysis buffer (50 mM Tris-HCl pH 7.5, 150 mM NaCl, 1 mM CaCl2, 1% Nonidet P-40, 0.5% sodium deoxycholate) supplemented with 1x cOmplete™ protease inhibitors cocktail (Roche, Oakville ON).

### BioID2 proximity-dependent biotinylation assay

HEK-293T cells were transfected for 5 h in Opti-MEM medium with pcDNA3.4 derivatives coding for BioID2_ProtC_ or BioID2-CLCF1_ProtC_ (0.4 µg) and p40_FLAG_ (3.6 µg) in the presence of polyethylenimine. A pcDNA3.4 derivative coding for eGFP was used as negative control. Transfected cells were incubated in complete DMEM medium overnight, and in Opti-MEM medium for 2 d. Biotin (50 µM) was added to the cell medium for the last 18 h of incubation. Cell supernatant was collected, and cells were lysed in RIPA buffer (50 mM Tris pH 7.5, 150 mM NaCl, 1% Nonidet P40, 0.5% sodium deoxycholate, 0.1% SDS) supplemented with 1x cOmplete™ protease inhibitors cocktail.

Supernatants of transfected HEK-293T cells were mixed with Triton X-100 (1% final concentration), and p40_FLAG_ was immunoprecipitated using anti-FLAG (Clone M2) affinity gel (Sigma-Aldrich, Oakville ON) according to manufacturer’s instructions. Proteins were eluted with SDS-PAGE sample loading buffer without β-mercaptoethanol, which was added subsequently before western blot analysis.

## Co-immunoprecipitation

CLCF1_Protc_ from cell lysates, or cell supernatants mixed with CaCl_2_ (1 mM final concentration) and Nonidet P40 (1% final concentration) was immunoprecipitated using anti-ProtC affinity matrix (Roche) according to manufacturer’s instructions, and eluted with the suggested elution buffer (20 mM Tris pH 7.5, 100 mM NaCl, 5 mM EDTA). Alternatively, CRLF1_FLAG_ from cell supernatants mixed with Triton X-100 (1% final concentration) was immunoprecipitated using anti-FLAG affinity gel according to manufacturer’s instructions, and eluted with SDS sample loading buffer without β-mercaptoethanol, which was added subsequently before western blot analysis.

To demonstrate that the CLCF1-p40 complex can form in the extracellular space, supernatants from HEK-293T cells transfected with CLCF1_ProtC_ and CRLF1_FLAG_, or p40 were mixed in equal parts and incubated at 37 °C for 24 h. The incubated supernatants were then mixed with CaCl_2_ and Nonidet P40, and CLCF1_ProtC_ was immunoprecipitated using anti-ProtC affinity matrix as described above.

To confirm the presence of a tripartite complex between CLCF1, CRLF1 and p40, cell supernatants were mixed with CaCl_2_ and Nonidet P40, and CLCF1_ProtC_ was immunoprecipitated using anti-ProtC affinity matrix as described above. Eluted fractions were then mixed with Triton X-100, and CRLF1_FLAG_ was immunoprecipitated using anti-FLAG affinity gel as described above.

## Western blot

All samples were subjected to SDS-PAGE, and electrotransferred to PVDF membranes. Membranes were probed with anti-ProtC (Clone HPC4; GenScript, Piscataway NJ), anti-FLAG (Clone M2; Sigma-Aldrich), or anti-p40 (R&D systems, Minneapolis MN), followed by an appropriate HRP-conjugated anti-IgG. Alternatively, membranes were probed with HRP-conjugated streptavidin (GE Healthcare, Chicago IL). Chemiluminescence signal was detected using an ImageQuant LAS 4000 (GE Healthcare), or autoradiography films. All images were processed using the ImageJ software.

### In silico docking analysis

Putative CLCF1-p40 complex structures were modelled using the AlphaFold3^53^ server (alphafoldserver.com). For each of the top 5 results returned by the server, we utilized the Surfaces method^54^ for the quantification of molecular interactions. Among the five AlphaFold models, model 3 was predicted to be the most stable. That model was subjected to a molecular dynamics energy minimization using the Yasara server^55^. Predicted Gibbs free energy change (ΔG) values were converted into dissociation constant (K_D_) values using the following equation: ΔG = R∙T∙ln(K_D_)​, where R represents the gas constant (1.9872 cal/mol·K) and T represents the temperature in Kelvin (K).

### Proximity ligation assay

Ba/F3 cells were treated with 3 µg/ml brefeldin A (Thermo Fisher Scientific) for 4 h, fixed with 2% formaldehyde at room temperature for 10 min, and permeabilized with 100% cold methanol on ice for 10 min. Protein interaction between CLCF1 and p40 was detected with the Duolink flowPLA violet detection kit (Sigma-Aldrich) according to manufacturer’s instructions, using 1 µg/test anti-CLCF1 mAb (Clone #138815; R&D systems) and 0.1 µg/test anti-p40 (Clone EPR20096-81; Abcam, Boston MA) as primary antibodies. Protein expression in Ba/F3 cells was confirmed using PE-conjugated anti-p40 (Clone C8.6; Thermo Fisher Scientific), and CF405M-conjugated anti-CLCF1. The anti-CLCF1 mAb was conjugated using the CF405M Mix-n-Stain antibody labeling kit (Sigma-Aldrich) according to manufacturer’s instructions. Fluorescence was quantified using a FacsCanto II flow cytometer (BD Biosciences, Franklin Lakes NJ). Data were analyzed using the FlowJo software (BD Biosciences).

### Binding assay

Ba/F3 transduced derivatives were incubated in PBS containing 0.1% BSA on ice for 1 h with 1 µg/ml of p40-linker-CLCF1 fusion protein from 10X concentrated HEK-293T cell supernatant. An equal volume of concentrated cell supernatant from HEK-293T cells transfected with a pcDNA3.4 derivative coding for eGFP was used as negative control. Recombinant biotinylated human CLCF1 produced in *E. coli* BL21 (DE3; Thermo Fisher Scientific) as previously described^13^ (1 µg/ml), or recombinant human IL23 (0.5 µg/ml; PeproTech) were used as controls. Protein binding to receptors was detected with PE-conjugated streptavidin (Thermo Fisher Scientific) for CLCF1, or biotinylated anti-p40 mAb (Clone C8.6; Thermo Fisher Scientific) followed with PE-conjugated streptavidin for p40-linker-CLCF1 and IL23. Non-viable cells were excluded using eFluor 506 Fixable Viability Dye (Thermo Fisher Scientific). Fluorescence was assessed using a FacsCanto II flow cytometer, and data were analyzed using the FlowJo software.

### Proliferation assay

Ba/F3 CNTFR, IL12R or IL23R (1 × 10^4^ cells/well in 96 well plates) were incubated with up to 100 ng/ml of p40-linker-CLCF1 or CLCF1-CRLF1 from 10X concentrated HEK-293T cell supernatant for 72 h in RPMI 1640 medium supplemented with 5% FBS. Recombinant human CLCF1 produced in *E. coli* BL21^13^ (up to 100 ng/ml) was used as a positive control for the proliferation of Ba/F3 CNTFR, while recombinant human IL12 or IL23 (up to 100 ng/ml; both from PeproTech) were used as positive controls for Ba/F3 IL12R and IL23R respectively. Proliferation was assessed using an alamarBlue fluorometric assay (BioRad, Hercules CA) and fluorescence at 590 nm was quantified with a Synergy H1 plate reader (BioTek, Winooski VT).

### STAT3 tyrosine phosphorylation assay

Ba/F3 CNTFR were serum starved for 4 h at 37 ºC in serum-free RPMI 1640 media. 1 × 10^6^ cells were stimulated at 37 °C for 15 min with 100ng/ml of p40, p40-linker-CLCF1 or CLCF1-CRLF1 from 10X concentrated HEK-293T cell supernatant. Cells were fixed for 15 min at 37 ºC with a fixation buffer (BioLegend), and permeabilized overnight at -20 ºC with a permeabilization buffer (BioLegend). Cells were stained with Alexa Fluor 488-conjugated anti-pSTAT3 mAb (pY705; BD Biosciences) for 30 min at 4 ºC. Non-viable cells were excluded using Zombie-aqua cell viability stain (BioLegend). Fluorescence was assessed using a BD LSRFortessa X-20 flow cytometer, and data were analyzed using the FlowJo software.

## Electronic supplementary material

Below is the link to the electronic supplementary material.

Supplementary Material 1

Supplementary Material 2

## Data Availability

All data generated or analysed during this study are included in this published article and its Supplementary Information files.
