# Supplementary material for: Cardiotrophin-like cytokine factor 1 forms a complex with IL12/IL23p40
Source: Sci Rep. 2025 Sep 30;15:33894. doi: 10.1038/s41598-025-08737-1 (PMC12485051; doi:10.1038/s41598-025-08737-1)
Supplement: Supplementary file 2 — Supplementary Material 2 [file 41598_2025_8737_MOESM2_ESM.docx]

**
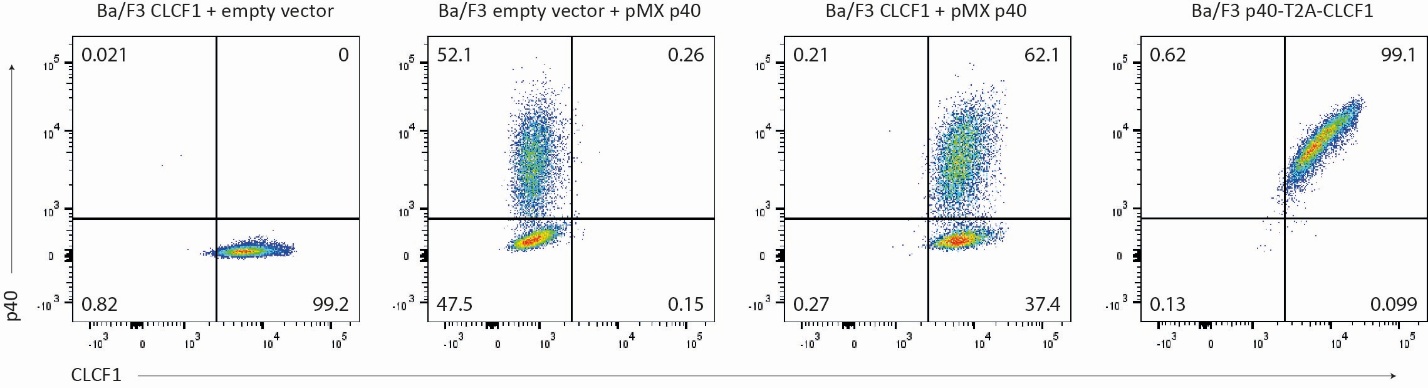
**

**Supplementary Figure 1. Confirmation of protein expression in the Ba/F3 cell derivatives used for the Duolink flowPLA proximity ligation assay.** Ba/F3 cells were transduced with pMX retroviruses coding for CLCF1, p40, or p40-T2A-CLCF1 cDNA. Representative flow cytometry profiles show CLCF1 and p40 expression.

**Supplementary Figure S2. Original Western blot images for Figure 1.** Original Western blot images in chemiluminescence (top) and incandescent light (bottom) for **(a)** Figure 1a, **(b)** Figure 1b, and **(c)** Figure 1d. Original Western blot images for the input blots of Figure 1d are shown in Supplementary Figure S3a.

**Supplementary Figure S3. Original Western blot images for Figure 2. (a)** Original Western blot images in chemiluminescence (top) and incandescent light (bottom) for Figure 2a. **(b-c)** Original Western blot images in autoradiography films for **(b)** Figure 2b, and **(c)** Figure 2c. Original Western blot images for the input blots of Figure 2b and 2c are shown in Supplementary Figure S3a. **(d)** Original Western blot images in chemiluminescence (top) and incandescent light (bottom) for Figure 2e.

**Supplementary Figure S4. Original Western blot images for Figure 3. (a-b)** Original Western blot images in chemiluminescence (top) and incandescent light (bottom) for **(a)** Figure 3a, and **(b)** Figure 3b.

**Supplementary Table S5. Predicted residue interactions in the CLCF1-p40 complex.** The residues implicated in the CLCF1-p40 interaction interface were predicted with an *in silico* docking analysis. The interactions are ordered from highest to lowest affinity, as represented by ΔG values.
